# Cardiac involvement in COVID-19 patients: mid-term follow up by cardiovascular magnetic resonance

**DOI:** 10.1186/s12968-021-00710-x

**Published:** 2021-02-25

**Authors:** Hui Wang, Ruili Li, Zhen Zhou, Hong Jiang, Zixu Yan, Xinyan Tao, Hongjun Li, Lei Xu

**Affiliations:** 1 Department of Radiology, Beijing Anzhen Hospital, Capital Medical University, No. 2, Anzhen Road, Chaoyang, Beijing, 100029 China; 2 Department of Rardiology, Beijing Youan Hospital, Capital Medical University, No. 8, Xi Tou Tiao Youanmen Wai, Fengtai, Beijing, 100069 China

**Keywords:** COVID-19, Cardiac involvement, Cardiac dysfunction, Cardiac magnetic resonance imaging

## Abstract

**Background:**

Coronavirus disease 2019 (COVID-19) induces myocardial injury, either direct myocarditis or indirect injury due to systemic inflammatory response. Myocardial involvement has been proved to be one of the primary manifestations of COVID-19 infection, according to laboratory test, autopsy, and cardiovascular magnetic resonance (CMR). However, the middle-term outcome of cardiac involvement after the patients were discharged from the hospital is yet unknown. The present study aimed to evaluate mid-term cardiac sequelae in recovered COVID-19 patients by CMR

**Methods:**

A total of 47 recovered COVID-19 patients were prospectively recruited and underwent CMR examination. The CMR protocol consisted of black blood fat-suppressed T2 weighted imaging, T2 star mapping, left ventricle (LV) cine imaging, pre- and post-contrast T1 mapping, and late gadolinium enhancement (LGE). LGE were assessed in mixed both recovered COVID-19 patients and healthy controls. The LV and right ventricle (RV) function and LV mass were assessed and compared with healthy controls.

**Results:**

A total of 44 recovered COVID-19 patients and 31 healthy controls were studied. LGE was found in 13 (30%) of COVID-19 patients. All LGE lesions were located in the mid myocardium and/or sub-epicardium with a scattered distribution. Further analysis showed that LGE-positive patients had significantly decreased LV peak global circumferential strain (GCS), RV peak GCS, RV peak global longitudinal strain (GLS) as compared to non-LGE patients (*p* < 0.05), while no difference was found between the non-LGE patients and healthy controls.

**Conclusion:**

Myocardium injury existed in 30% of COVID-19 patients. These patients have depressed LV GCS and peak RV strains at the 3-month follow-up. CMR can monitor the COVID-19-induced myocarditis progression, and CMR strain analysis is a sensitive tool to evaluate the recovery of LV and RV dysfunction.

## Introduction

Coronavirus disease 2019 (COVID-19) is a nascent pandemic. Until July 20, 2020, 14,353,494 confirmed cases, including 603,703 deaths, were reported to the World Health Organization [1]. Data from previous studies suggested that acute cardiac injury occurred in 20% COVID-19 patients [2]. In hospitalized patients, the cardiac injury was up to 30% and caused 40% deaths [3–6]. The mechanisms of cardiac injury are direct myocarditis (direct myocardial infection by SARS-CoV-2) or indirect factors, such as cardiac stress due to respiratory failure, indirect injury from systemic inflammatory response-cytokine release syndrome, stress cardiomyopathy, or a combination of all these factors [6–9].

Cardiovascular magnetic resonance (CMR) can visualize and quantify heart volume and function and characterize the myocardial tissue; thus, it has been used as a gold standard non-invasive imaging tool in cardiovascular medicine [10]. A recent single-center study from Wuhan demonstrated that more than half of the recovered COVID-19 patients sustain cardiac edema, fibrosis, and impaired right ventricle (RV) contractile function [11]. However, in this small-sample retrospective study, only patients with reported cardiac symptoms were included. The middle-term outcome of cardiac involvement in COVID-19 patients is yet unknown. Thus, the present study aimed to evaluate mid-term cardiac sequelae in recovered COVID-19 patients by CMR.

## Methods

### Study design and participants

For this prospective, single-center study, we recruited consecutive COVID-19 patients from May 8 to July 20, 2020. The inclusion criteria were as follows: (1) confirmed to have SARS-CoV-2 infection, (2) recovered from COVID-19 and discharged from the hospital for 12 weeks, (3) agreed to participate and signed informed consent. The exclusion criteria were as follows: (1) pacemaker placement, (2) uncontrolled hypertension, (3) coronary artery disease (evidence of coronary artery stenosis > 50%) or previous myocardial infarction, (4) moderate to severe valvular dysfunction, (5) previous atrial fibrillation, (6) previous heart failure, (7) previous myocarditis, (8) known cardiomyopathy, (9) severe renal insufficiency (creatinine clearance rate < 30 mL/min/1.73 m^2^, (10) unable to cooperate with breath-holding and cannot undergo CMR examination, (11) pregnancy, (12) not suitable due to other factors.

Age- and sex-matched healthy controls, who previously underwent CMR in our hospital were selected from a health screening database. All the controls had a normal electrocardiogram (ECG), echocardiography, and CMR and did not present any cardiovascular disease or systemic inflammation. The present study was approved by the local institutional review board (KS2020001), and informed consent was obtained from all patients.

### CMR scanning protocol

All patients underwent CMR on a 3 T CMR scanner (Ingenia CX, Philips Healthcare, Best, The Netherlands). The CMR protocol consisted of black blood fat-suppressed T2 weighted imaging (T2w), T2 star mapping, left ventricle (LV) cine imaging including four chambers, two-chamber, short axis, pre- and post-contrast T1 mapping, and late gadolinium enhancement (LGE).

Black blood T2w imaging was performed in short axis using multishot turbo spin echo (TSE) sequence with time of repetition (TR) = 2 heart beat periods, time of echo (TE) = 75 ms, voxel size = 1.7 × 1.7 × 8 mm^3^, field of view (FOV) = 380 × 380 mm^2^, flip angle (FA) = 90°, and acceleration factor = 2. T2 star mapping was carried out by a turbo field echo (TFE) sequence with 15 TEs from 1.15 to 16 ms, TR = 29 ms, voxel size = 1.8 × 1.8 × 15 mm^3^, FOV = 300 × 300 mm^2^, FA = 25°, and acceleration factor = 3. The cine scanning was conducted by a balanced steady state-free precession (bSSFP) sequence, with TR/TE = 3.0/1.52 ms, FA = 45°, voxel size = 1.8 × 1.8 × 8 mm^3^, FOV = 270 × 270 mm^2^, and acceleration factor = 1.5 or 3.6 depending on the number of slices acquired per breath hold. For short axis cine imaging, 9 slices were acquired to cover the entire LV. Modified Look-locker inversion recovery (MOLLI) acquisition scheme was applied for both pre- and post-contrast T1 mapping using single-shot bSSFP sequence with TR/TE = 3.3/1.5 ms, voxel size = 2 × 2 × 15 mm^3^, FOV = 300 × 300 mm^2^, FA = 20°, and acceleration factor = 2. LGE imaging was implemented 10 min after intravenous administration of contrast medium (0.2 mmol/kg; Magnevist gadopentetate dimeglumine, Bayer Healthcare, Bayer, Berlin, Germany) using a phase-sensitive inversion-recovery (PSIR) TFE sequence with TR/TE = 6.1/3.0, voxel size = 1.6 × 1.9 × 8 mm^3^, FOV = 350 × 350 mm^2^, FA = 25°/5°, and acceleration factor = 2. All the protocols were executed using ECG trigger and breath holding.

### CMR images analysis

Both COVID-19 and healthy subject data were intermingled. And anonymized images were evaluated by 3 radiologists (ZZ, HW and LX with 5, 8 and 12 years of CMR diagnosis experience, respectively). The LGE lesion was quantified using full width at half-maximum method [12]. The visual presence and different patterns (epicardial, mid-wall, or transmural) on the LGE images were assessed by three radiologists independently. Any discrepancies were resolved by consensus. The ratio between the LGE volume and the total LV myocardium volume (LGE/myocardium) in the LGE-positive patients was calculated.

Whole LV myocardium region (including regions of LGE lesion) were delineated on the native T1 mapping and gobal T1 values were computed using cvi42 software (Circle Cardiovascular Imaging Inc., Calgary, Alberta, Canada).

The LV and RV function and LV mass were assessed based on the short-axis cine images using cvi42 software (Circle Cardiovascular Imaging). Endocardial and epicardial borders, with papillary muscles excluded from volumes, were identified automatically by the software and amended by a radiologist (HW). LV and RV range were defined from the planes of the mitral valve and tricuspid valve to the apex, respectively, on 4-chamber cine images in both diastolic and systolic phases. Short-axis images were divided into size-based equiangular segments with RV-LV junction as the reference point. LV and RV function parameters, end-diastolic volume (EDV), end-systolic volume (ESV), stroke volume (SV), cardiac output (CO), ejection fraction (EF), and LV mass were calculated automatically. All volumes and masses were normalized to the body surface area (BSA).

Three-dimensional (3D) global radial strain (GRS), global circumferential strain (GCS), and global longitudinal strain (GLS) of LV and RV were obtained using cvi42 (Circle Cardiovascular Imaging). The end-diastolic phase served as the reference. Contours of endo- and epicardial myocardium of short-axis, as well as the 2-, 3-, 4-chamber long-axis cine images, were drawn by two radiologists (HW and ZZ respectively). Patients were further divided into two subgroups based on the presence of LGE.

### Statistical analysis

All data were analyzed using SPSS (version 25.0, Statistical Package for the Social Sciences, International Business Machines, Inc., Armonk, New York, USA). Normally distributed continuous variables were expressed as mean ± standard deviation. Two-tailed one-way ANOVA was used to analyze the differences between LGE, non-LGE, and normal control groups. Categorical variables were expressed as counts and percentages. T-test was used to compare the means with normal distribution, and the Mann–Whitney U test was used to compare the variables with non-normal distribution between LGE and non-LGE groups. χ^2^ test was used to explore the statistical significance of CMR parameters among LGE, non-LGE, and normal control groups. A two-sided *p* < 0.05 was considered as statistically significant.

Inter- and intra-observer intraclass coefficients (ICC) was used to assess reproducibility of strain measurements. The same investigator (HW) measured the 3D global strain of LV and RV in the all 75 studies on two occasions, separated by an interval of 2 months to assessment of intra-observer reproducibility. And a second investigator (ZZ) also evaluated all 75 subjects to assess inter-observer reproducibility.

## Results

### Population characteristics

From May 8 to July 20, 2020, 47 patients who recovered from COVID-19 were recruited and underwent CMR. One patient was excluded because of moderate tricuspid regurgitation, one was excluded because of hypertrophic cardiomyopathy, and one was excluded because of hypertensive cardiomyopathy. The resulting 44 recovered patients and 31 healthy controls were included in this study. The clinical characteristics of COVID-19 patients are reported in Table 1. The average age of COVID-19 patients was 47.6 ± 13.3 years, and the cohort comprised of 19 (43.2%) men. Among the 31 normal controls, 19 were male, which matched with the COVID-19 group. Althoug only 12 female healthy controls were included, there was no significant difference in sex distribution among patients with LGE-positive, patients with LGE-negative and normal controls (P > 0.05)(Table 2).

**Table 1:** Clinical characteristics, laboratory measurements, complications during hospitalization and treatment before discharge of recovered COVID-19 patients

Characteristic	Total COVID-19 patients (n = 44)	LGE (N = 13)	Non-LGE (N = 31)	P-value
Age (years)	47.6 ± 13.3	53.2 ± 14.5	45.2 ± 12.43	0.07
Gender	19 (43.2%)	4 (30.8%)	15 (48.4%)	0.34
BSA (kg/m^2^)	1.76 ± 0.19	1.73 ± 0.17	1.77 ± 0.2	0.63
Heart rate (bpm)	66.2 ± 11.7	64.1 ± 6.8	67.0 ± 13.2	0.32
Time between hospital discharge and CMR (days)	102.5 ± 20.6	100.8 ± 20.3	103.3 ± 21.0	0.72
Regular exercise	10 (22.7%)	3 (23.1%)	7 (22.6%)	1.0
Smoke	1 (2.3%)	0	1 (3.2%)	NA
Alcohol	3 (6.8%)	1 (7.7%)	2 (6.5%)	1.0
NYHA I/II/III/IV	21/13/8/2	4/5/3/1	17/8/5/1	0.52
Clinical COVID-19 pneumonia types, moderate/severe/critical	32/11/1	7/5/1	25/6/0	0.10
Comorbidities				
Hypertension	11 (25.0%)	5 (38.5%)	6 (19.4%)	0.18
Diabetes mellites	8 (18.2%)	2 (15.4%)	6 (19.4%)	1.0
Hyperlipidemia	16 (36.4%)	3 (23.1%)	13 (41.9%)	0.31
Chronic obstructive pulmonary disease	0	0	0	NA
Cerebrovascular disease	0	0	0	NA
Chronic renal diseases	0	0	0	NA
Hepatitis B	2 (4.6%)	1 (7.7%)	1(3.2%)	0.51
Laboratory findings				
CPK (u/l)*	69 (14–141)	57 (45–167)	76 (45–142)	0.85
CKMB (ng/ml)*	0.33 (0.18–0.64)	0.32 (0.11–0.73)	0.33 (0.19–0.64)	0.86
MYO (ng/ml)	56.8 ± 42.7	66.9 ± 59.7	52.6 ± 33.6	0.31
TnI (ng/ml)*	0.02 (0.01–0.02)	0.02 (0.01–0.04)	0.01 (0.01–0.02)	**0.04**
CRP (mg/l)	43 ± 43	55 ± 54	38 ± 37	0.24
Potassium (mmol/l)	3.8 ± 0.3	3.7 ± 0.3	3.8 ± 0.3	0.24
Calcium (mmol/l)	1.3 ± 0.2	1.3 ± 0.3	1.3 ± 0.2	0.33
Other complications during hospitalization				
cardiac arrythmia	0	0	0	NA
Renal injury	4 (9.1%)	1 (7.7%)	3 (9.7%)	1.0
Liver injury	19 (43.2%)	5 (38.5%)	14 (45.2%)	0.68
Treatment before discharge				
Antiviral therapy	24 (54.5%)	9 (69.2%)	15 (48.4%)	0.21
Antibiotic therapy	12 (27.3%)	5 (38.5%)	7 (22.6%)	0.28
Corticosteroids	13 (29.6%)	6 (46.2%%)	7 (22.6%)	0.12
Non-invasive ventilation of high-flow nasal cannula oxygen	10 (22.7%)	5 (38.5%)	5 (16.1%)	0.11
Intravenous immunoglobulin	2 (4.6%)	1 (7.7%)	1 (3.2%)	0.51
ACEI/ARB	2 (4.6%)	1 (7.7%)	1 (3.2%)	0.51

Boldface characters indicate *p* < 0.05

*ACEI* angiotensin converting enzyme inhibitor, *ARB* angiotensin receptor blocker, *BSA* body surface area, *CMR* cardiovascular magnetic resonance, *CPK* creatine phosphokinase, *CRP* C-reactive protein, *LGE* late gadolinium enhancement, *MYO* myohemoglobin, *NYHA* New York Heart Association, *TnI* troponin I

^*^Median (IQR)

**Table 2:** CMR parameters of recovered COVID-19 patients and normal controls

Characteristic	Total (n = 75)	COVID-19 patients (n = 44)		Healthy controls (n = 31)	P-Value*	Adjusted P-value**	Adjusted P-value^***^	Adjusted P-value^****^
		LGE-positvie (n = 13)	LGE-negative (n = 31)					
Age (years) Average ± SD	47.4 ± 12.3	53.2 ± 14.5	45.2 ± 12.3	47.1 ± 11.0	0.15	0.05	0.14	0.55
Gender, Male	38 (50.7%)	4 (30.8%)	15 (48.4%)	19 (61.3%)	0.17	0.34	0.06	0.31
BSA (kg/m^2^)	1.8 ± 0.2	1.7 ± 0.2	1.8 ± 0.2	1.8 ± 0.2	0.38	0.59	0.20	0.33
Heart rate (bpm)	66.4 ± 10.6	63.8 ± 6.6	67.2 ± 13.4	66.6 ± 8.8	0.61	0.33	0.43	0.81
LVEDV	129.1 ± 25.5	120.9 ± 26.8	130.9 ± 27.8	130.8 ± 22.7	0.45	0.24	0.25	0.99
LVESV	48.5 ± 12.6	42.8 ± 9.7	49.8 ± 13.3	49.7 ± 12.6	0.20	0.10	0.10	0.98
LVSV	80.6 ± 17.1	78.0 ± 21.0	81.1 ± 16.6	81.1 ± 17.1	0.84	0.58	0.60	0.98
LVEF	62.5 ± 5.7	64.3 ± 5.9	62.2 ± 4.4	62.0 ± 6.7	0.46	0.28	0.23	0.88
LVCO	5.3 ± 1.2	4.9 ± 1.1	5.3 ± 1.1	5.4 ± 1.2	0.44	0.25	0.23	0.93
LVCI	3.0 ± 0.6	2.8 ± 0.5	3.0 ± 0.6	3.0 ± 0.6	0.54	0.27	0.50	0.59
LV mass	76.7 ± 15.1	72.5 ± 14.5	74.3 ± 15.4	80.7 ± 14.7	0.14	0.72	0.10	0.97
LVEDV/BSA	72.4 ± 11.7	69.3 ± 10.6	73.8 ± 12.7	72.4 ± 11.2	0.52	0.25	0.43	0.63
LVESV/BSA	27.1 ± 6.4	24.7 ± 5.1	27.8 ± 6.4	27.5 ± 6.8	0.31	0.14	0.19	0.85
LVSV/BSA	45.3 ± 7.9	44.6 ± 8.6	46.0 ± 7.8	44.8 7.9	0.81	0.60	0.93	0.57
LVCO/BSA	3.0 ± 0.6	2.8 ± 0.5	3.0 ± 0.6	3.0 ± 0.6	0.49	0.24	0.44	0.59
LV mass/BSA	42.9 ± 5.6	41.6 ± 5.6	41.8 ± 5.4	44.4 ± 5.7	0.13	0.91	0.13	0.07
RVEDV	128.8 ± 31.5	118.1 ± 32.8	129.6 ± 34.2	133.5 ± 27.9	0.34	0.31	0.14	0.54
RVESV	54.4 ± 18.3	47.3 ± 15.0	56.7 ± 21.3	55.0 ± 16.1	0.30	0.13	0.20	0.73
RVSV	74.5 ± 18.7	70.8 ± 22.6	72.1 ± 19.2	78.5 ± 16.1	0.30	0.84	0.22	0.18
RVEF	58.1 ± 7.7	59.5 ± 8.6	56.6 ± 8.3	59.1 ± 6.5	0.35	0.26	0.89	0.20
RVCO	4.9 ± 1.2	4.5 ± 1.3	4.8 ± 1.3	5.2 ± 1.0	0.16	0.46	0.08	0.18
RVCI	2.8 ± 0.6	2.6 ± 0.7	2.7 ± 0.7	2.8 ± 0.5	0.40	0.40	0.18	0.51
RVEDV/BSA	71.3 ± 13.8	67.6 ± 14.4	72.4 ± 15.2	71.81 ± 12.23	0.569	0.30	0.36	0.87
RVESV/BSA	30.4 ± 9.1	27.0 ± 7.0	31.8 ± 10.3	30.5 ± 8.4	0.30	0.12	0.25	0.61
RVSV/BSA	41.5 ± 9.0	40.5 ± 10.9	40.8 ± 9.5	42.6 ± 7.9	0.68	0.94	0.50	0.44
RVCO/BSA	2.7 ± 0.6	2.6 ± 0.7	2.7 ± 0.7	2.9 ± 0.5	0.32	0.50	0.15	0.33
Native T1(ms)	–	1286 ± 60	1253 ± 55		0.09			
3D-Strain								
LV peak GRS	42.6 ± 19.5	40.5 ± 18.7	46.3 ± 23.0	39.8 ± 15.8	0.39	0.37	0.91	0.19
LV peak GCS	− 17.5 ± 6.5	− 15.1 ± 10.3	− 16.7 ± 6.9	− 19.4 ± 3.0	0.08	0.45	**0.04**	0.10
LV peak GLS	− 12.7 ± 3.3	− 11.9 ± 3.9	− 12.7 ± 3.6	− 13.1 ± 2.8	0.58	0.47	0.30	0.68
RV peak GRS	31.6 ± 12.6	31.4 ± 14.5	28.8 ± 11.1	34.4 ± 10.4	0.16	0.49	0.42	0.06
RV peak GCS	− 11.9 ± 4.2	− 9.4 ± 3.4	− 12.1 ± 4.0	− 12.9 ± 4.3	**0.04**	**0.05**	**0.01**	0.46
RV peak GLS	− 11.3 ± 4.0	− 7.8 ± 4.0	− 12.9 ± 3.0	− 11.3 ± 3.9	**0.00**	**0.003**	**0.00**	0.08

Boldface characters indicate p (adjusted p) < 0.05

*BSA* body surface area, *CI* cardiac index, *CO* cardiac output, *EDV* end-diastolic volume, *EF* ejection fraction, *ESV* end-systolic volume, *GCS* global circumferential strain, *GLS* global longitudinal strain, *GRS* global radial strain, *LGE* late gadolinium enhancement, *LV* left ventricle, *RV* right ventricle, *SV* stroke volume

^*^P-value is for patients with LGE-positive versus patients with LGE-negative versus normal controls

^**^Adjusted P-value: Statistical difference between patients with and without LGE on CMR imagings

^***^P-value: Statistical difference between patients with LGE-positive on CMR imagings and normal controls

^****^P-value: Statistical difference between patients with LGE-negative on CMR imagings and normal controls

The average duration from discharge from the hospital to CMR examination was 102.5 ± 20.6 days. A quarter of the patients performed regular exercise, only one patient smoked, and three drank small amount alcohol every day (less than 25 mg per day). New York Heart Function Classification from I to IV was 21, 13, 8, 2, respectively. According to the Diagnosis and Treatment Protocol of Novel Coronavirus issued by the National Health Commission of the People’s Republic of China [13], the moderate, severe, and critically ill types COVID-19 pneumonia were 32 of 44 (72.7%), 11 (25.0%), and 1 (2.3%), respectively; 11 (25.0%) patients had hypertension, 8 (18.2%) patients had diabetes, and 16 (36.4%) patients had hyperlipidemia before COVID-19. Non-patients had chronic obstructive pulmonary disease, cerebrovascular disease, or chronic renal diseases. Moreover, 2 (4.6%) patients had hepatitis B before COVID-19. Twenty-four (54.5%) patients were administered antiviral (Kaletra, Arbidol, oseltamivir, and interferon), 12 (27.3%) patients were given antibiotic (moxifloxacin, cefixime, and cefuroxime) and 13 patients were given corticosteroid therapy, while 10 (22.7%) patients were given high-flow oxygen support, 2 (4.6%) patients were administered intravenous immunoglobulin, and 2 (4.6%) were given angiotensin-converting enzyme inhibitors (ACEI) or angiotensin receptor blockers (ARB) (Table 1).

Among all 44 recovered patients, normal ECGs were revealed in 43 patients. ST segment elevation in leads II, III, AVF and prolonged PR interval during initial diagnosis was demonstrated in one patient. Echocardiography was performed in 5 patients, all showed a small amount of mitral or/and tricuspid regurgitation.

### Laboratory results

During hospitalization, troponin I (TnI), creatine phosphokinase (CPK), Creatine phosphokinase-MB (CPK-MB), myohemoglobin (MYO), C-reactive protein (CRP), serum potassium, and serum calcium were measured in all patients at least one time. The median (interquartile range, IQR) or average ± standard deviation (SD) results of the highest value were as follows: TnI: 0.02 (0.01–0.02) ng/mL, CK: 69 (13.7–141.3) U/L, CKMB: 0.33 (0.18–0.64) ng/mL, MYO: 56.8 ± 42.7 ng/mL, CRP: 43.0 ± 42.9 mg/L, serum potassium: 3.8 ± 0.3 mmol/L, serum calcium: 1.3 ± 0.2 mmol/L. TnI in LGE group and non-LGE group were 0.02 (0.01–0.04) and 0.01 (0.01–0.02), respectively, with significant difference between the two groups (p < 0.05). (Table 1).

## CMR results

### LGE and T1 mapping

**Fig. 1:**
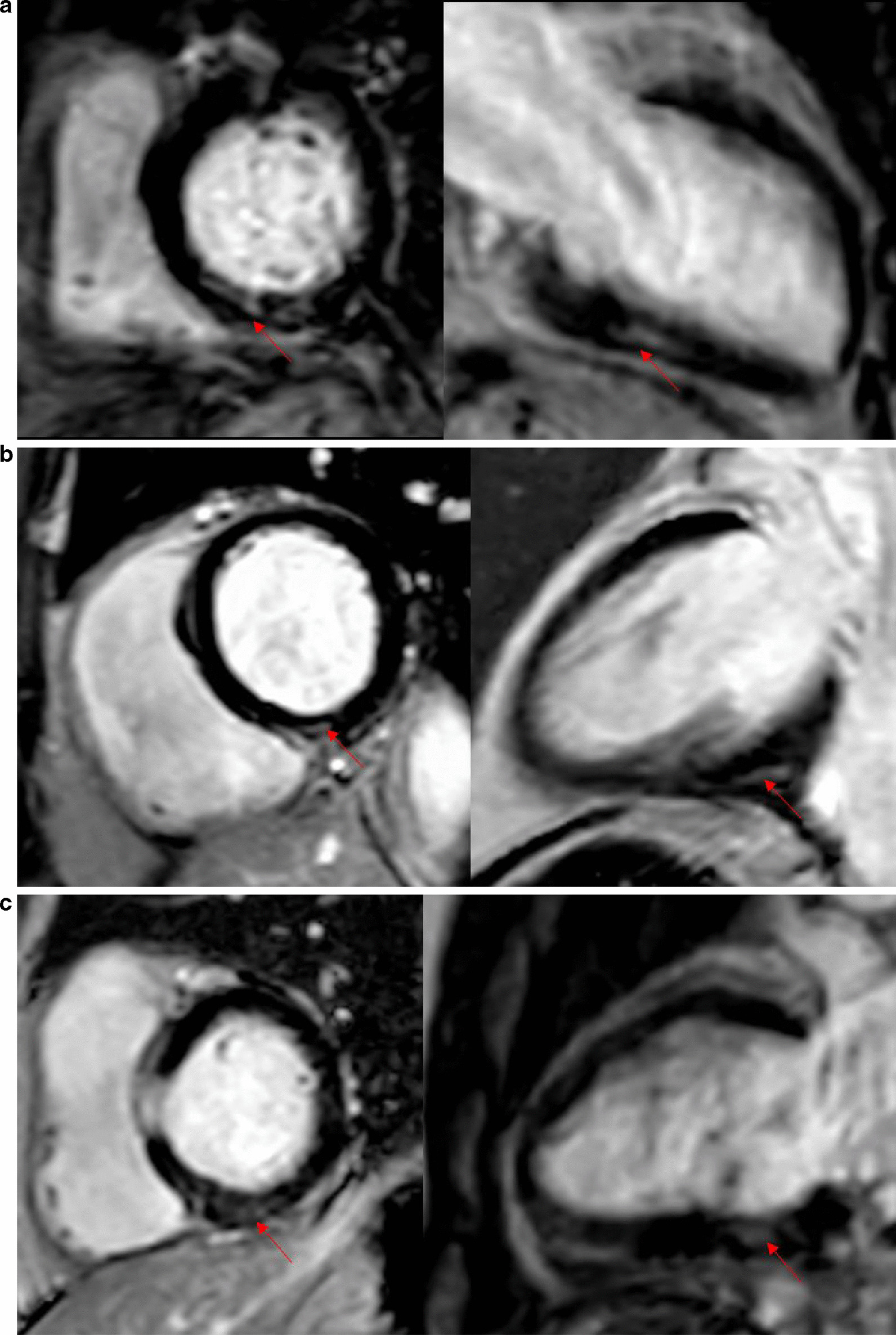

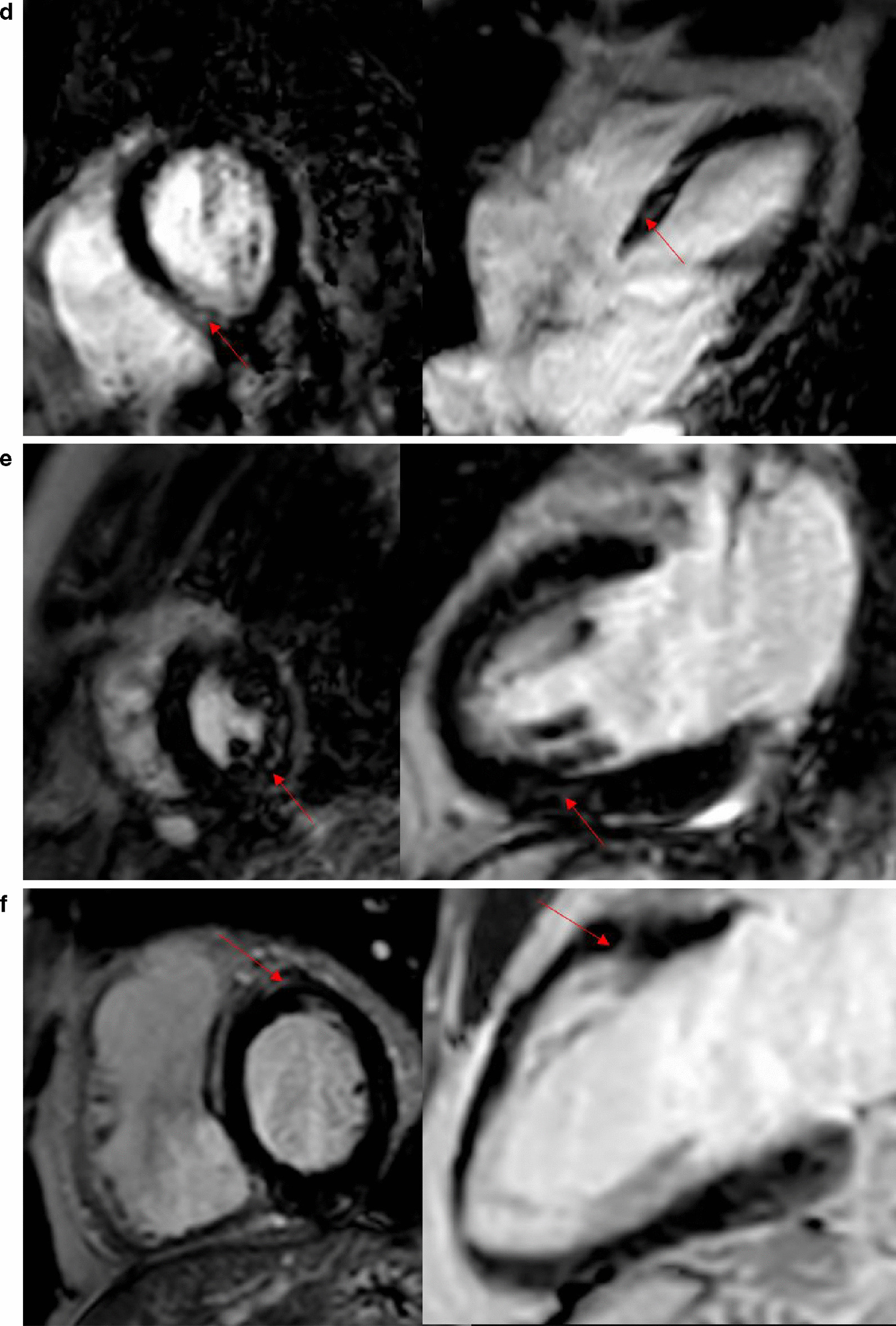

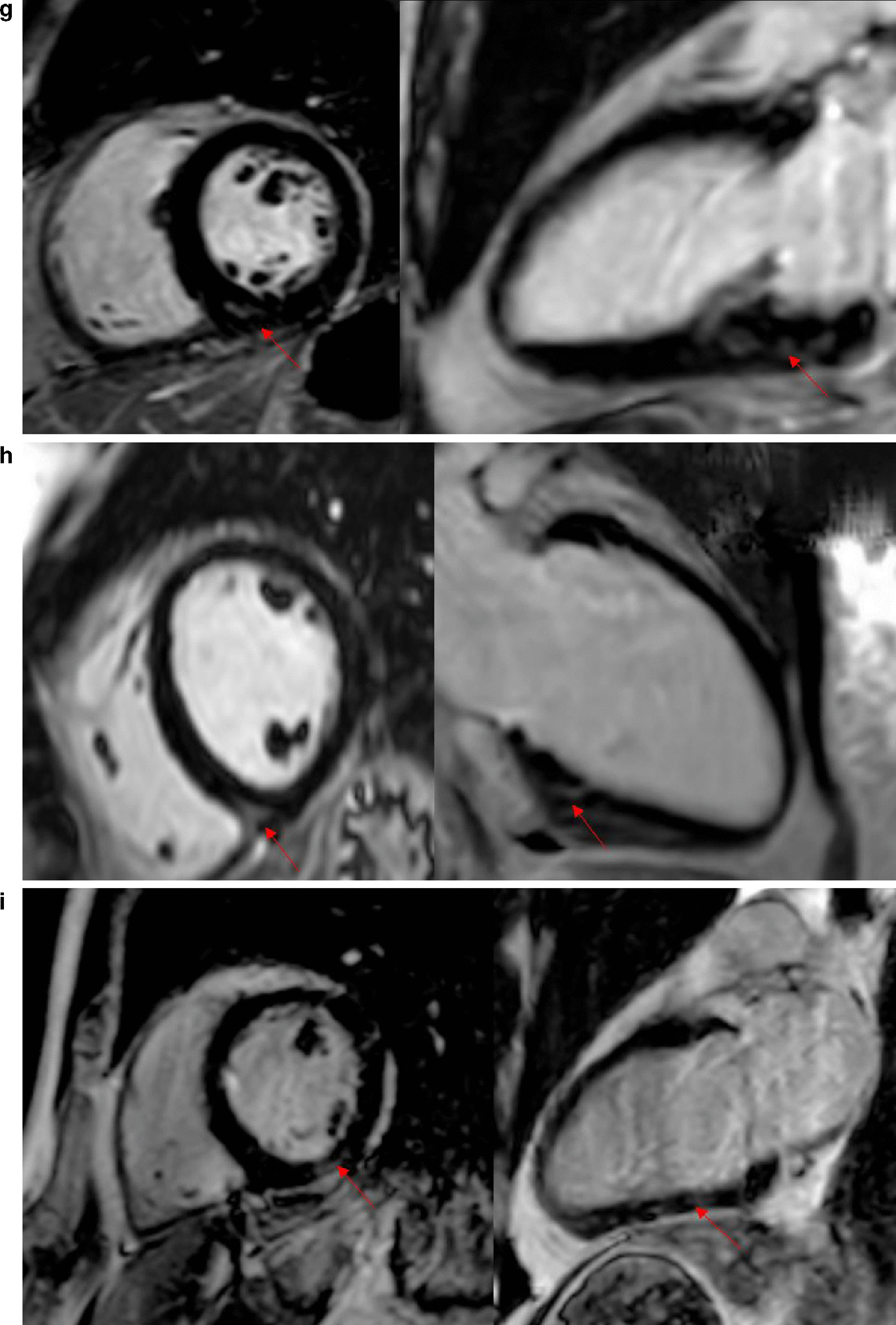

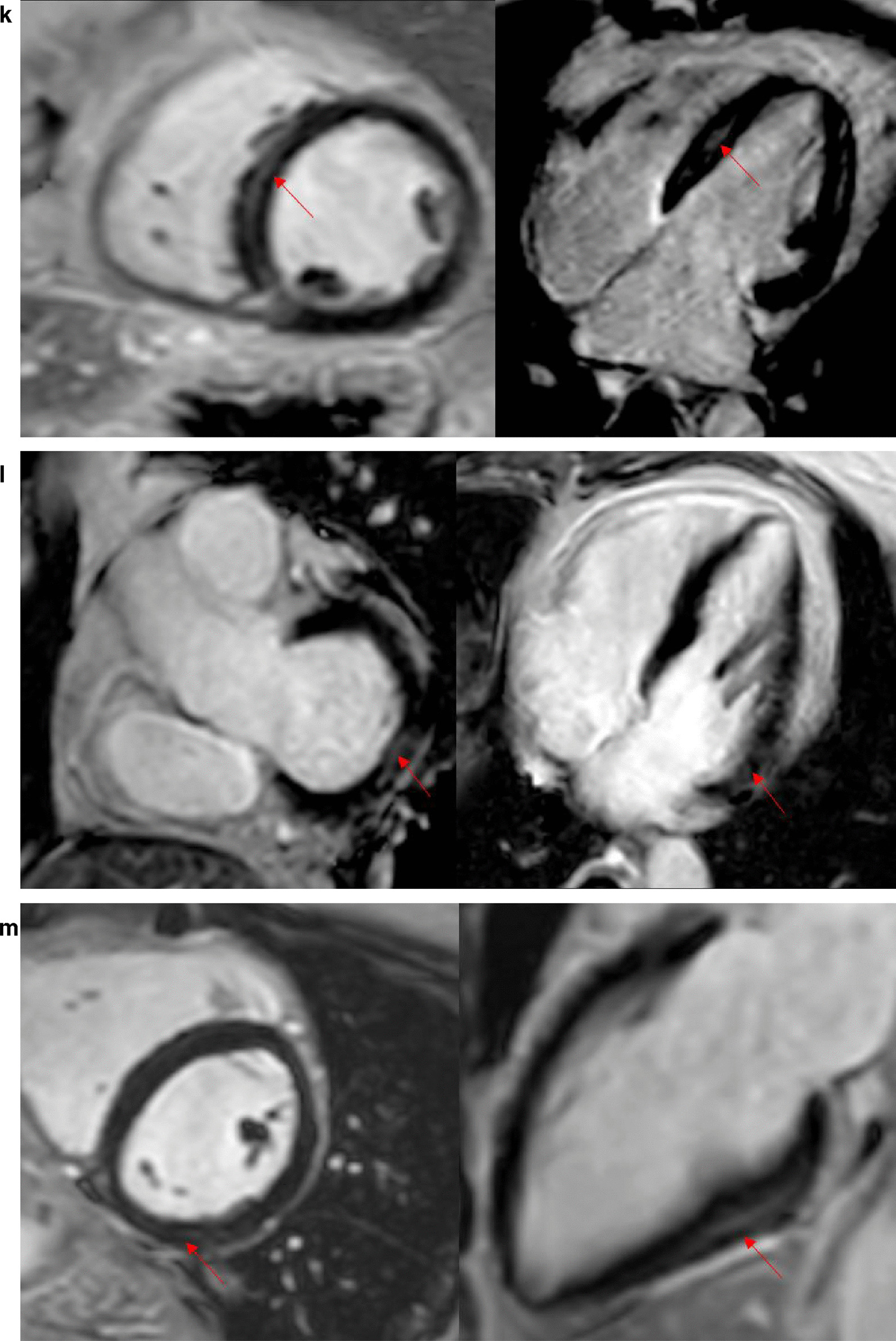
Illustration of all13 late gadolinium enhancement (LGE) positvie patients’ myocardial injury. **a**–**m** represents case 1–13, respectively. One short axis and orthogonal long-axis phase sensitive inversion recovery (PSIR) images show LGE (arrow) for each patient. *PSIR* phase-sensitive inversion recovery, *LGE* late gadolinium enhancement

**Fig. 2:**
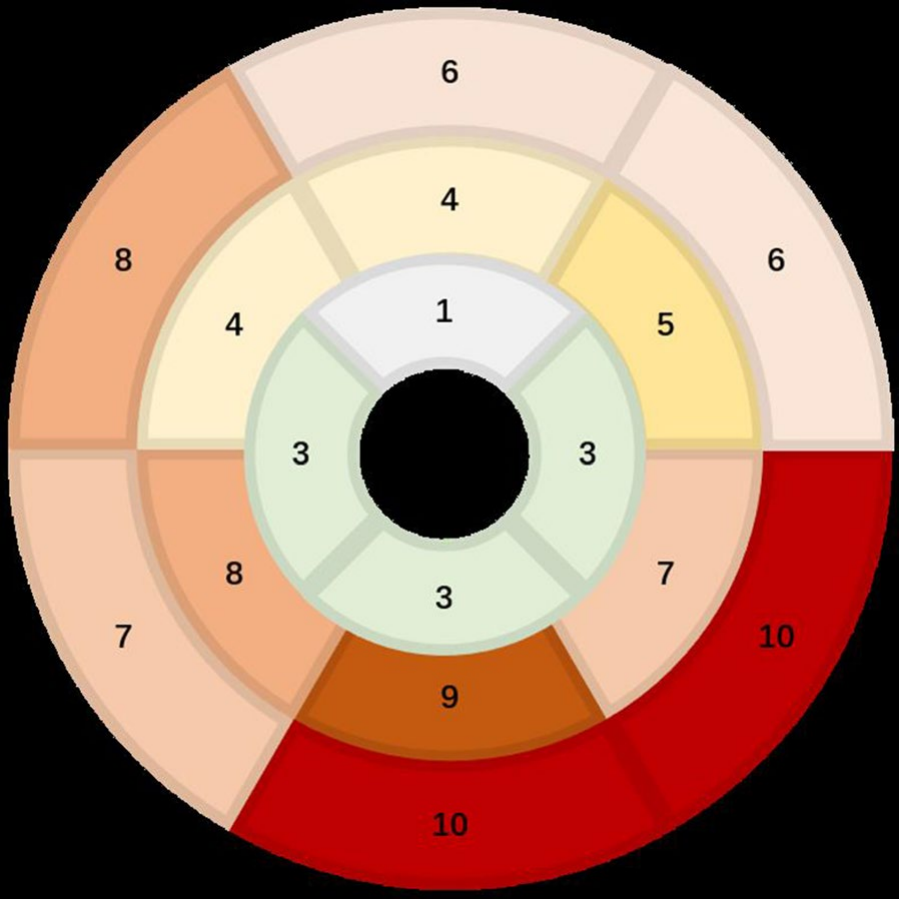
Dominant distribution of myocardial LGE segments in recovered COVID-19 patients. Number of myocardial LGE distributed in American Heart Association 16 segment model in all the 13 patients

No myocardium hemorrhage was observed in any patient; however, LGE was detected in 13 patients (29.5%). All of the LGE-positive patients were from the COVID-19 group, no LGE was observed any of the healthy controls. All LGE lesions were located in the middle myocardium and/or sub-epicardium with a scattered distribution (Fig. 1). Case 1 was the only patient with abnormal ECG findings. Among a total of 208 myocardial segments in 13 patients, most LGE lesions were located at the inferior and inferior-lateral segments at the base and mid-chamber. The Bull’s eye illustration (Fig. 2) shows us the number of myocardial LGE distributed in the American Heart Association 16 segments’ model for all 13 patients. The inferior wall and inferior-lateral wall of the basal segment was the most frequently involved segment (10/12 patients). The median of LGE/myocardium ratio was 1.7% (1.1–3.0%).

Global native T1 showed no significant difference between COVID-19 patients with and without LGE (1286 ms ± 60 ms vs 1253 ms ± 55 ms, P > 0.05).

### LV/RV morphological, function, and strain analysis

Table 2 shows the values for global LV and RV morphological and functional parameters and the measurement of the 3D global CMR feature-tracking deformation parameters. No significant difference was detected in the LV and RV morphological parameters (EDV, ESV, and mass) among COVID-19 patients with and without LGE and normal controls. Although no significant difference was detected in the LV and RV traditional function parameters—EF, CO, cardiac index (CI), SV; LV peak GCS was decreased in COVID-19 patients with visual LGE (− 15.1 ± 10.3) on CMR images as compared to healthy controls (− 19.4 ± 3.0) (*p* < 0.05). Both peak 3D GCS and GLS of RV in COVID-19 patients with LGE (− 9.4 ± 3.4 and − 7.8 ± 4.0, respectively) were significantly decreased as compared to COVID-19 patients without LGE (− 12.1 ± 4.0 and − 12.9 ± 3.0, respectively) and normal control (− 12.9 ± 4.3 and − 11.3 ± 3.9, respectively) (both *p* < 0.05). However, no difference (peak 3D GCS and GLS of RV) was detected between COVID-19 patients without LGE and the normal controls (*p* > 0.05).

Intra-observer ICC of LV peak 3D-GCS, RV peak 3D-GCS and RV peak 3D-GLS were 0.93, 0.92 and 0.77 respectively. Inter-observer ICC of LV peak 3D-GCS, RV peak 3D-GCS and RV peak 3D-GLS were 0.82, 0.70 and 0.76 respectively.

## Discussion

The present prospective study among recovered COVID-19 patients found that ~ 30% had CMR evidence for myocardial injury (manifested as LGE) at 3-month follow up. LGE-positive patients had a decrease in the LV GCS compared to healthy controls and a significant decrease in the RV GCS and GLS as compared to non-LGE COVID-19 patients.

Myocardial involvement has been proved to be one of the primary manifestations of COVID-19 infection by laboratory, autopsy, and CMR [14–16] as compared to other members of the coronavirus family [17]. A recent study demonstrated that in the recovered COVID-19 patients with cardiac symptoms, 54% had myocardium edema, and 31% had LGE [11]. The persistence of visual LGE at 3-month follow-up reflecting necrosis or scar (fibrosis) might be caused by COVID-19 and needs to be elucidated further [18]. Yet, visual LGE indicated that up to 30% COVID-19 patients have irreversible myocardial injury [19, 20], which is consistent with the findings of the previous study and can confirm one of the main mechanisms of COVID-19 induced direct myocardial injury—myocarditis [21–23]. In addition, the presence of LGE has been proven to be an independent predictor of all-cause mortality and cardiac mortality in myocarditis [24, 25]. Since the LGE/myocardium ratio was small, the cardiac status of COVID-19 patients with LGE needs to be closely monitored.

We found all of the LGE lesions to be located in the middle myocardium and/or sub-epicardium, wherein the fibers are oriented transversely, and the torque enhances shortening in the circumferential direction [26, 27]. This may be the reason for LV 3D GCS to be oriented along the perimeter in short axis view [26] and decreased in the COVID-19 patients with LGE. This finding prompts us to focus on the LV circumferential contraction dysfunction in these patients in addition to impaired RV function.

Impaired RV function in COVID-19 survivors has been demonstrated by echocardiography and CMR [11, 28, 29]. RV strain has been recommended to assess the RV function in clinical scenarios with suspected RV dysfunction [30]. Although our results showed that the RV traditional morphological and function parameters are in a normal range, the RV strain in COVID-19 patients with LGE was significantly decreased as compared to those without LGE and healthy controls. This phenomenon indicated that COVID-19 patients with LGE still had RV dysfunction, which could be detected by CMR strain analysis. COVID-19 results in acute respiratory distress syndrome (ARDS) [31] and is frequently associated with RV dysfunction, increased pulmonary resistance [32], increased values of systolic pulmonary arterial pressure, increased RV afterload [33, 34], severe hypoxia, oxidative stress, and increased myocardial oxygen demand induced by ARDS [35].

According to a recent systematic echocardiographic study, the most frequent abnormality induced by COVID-19 was RV dilation with or without dysfunction [32]. However, our results only showed RV dysfunction in patients with LGE. No RV morphological (EDV and ESV) difference was detected at the 3-month follow up, which might be attributed to improved pneumonia; subsequently, the RV rapidly returns to normal size while the RV strain is still decreased. Global native T1 value in both LGE-positive and LGE-negative COVID-19 patients were elevated compared with [1122 (1100, 1143) ms and 1122 ± 57 ms] healthy subjects, performed at Philips 3 T CMR with MOLLI sequence, previously reported by Gottbrecht et al. [36] and Clotilde Roy et al [37].

### Limitations

The current study has limitations. First, the lack of baseline pre-COVID-19 CMR examination limits the evaluation of the progress of heart involvement. Second, the sample size was relatively small. Third, the majority of the recruited patients had moderate and severe COVID-19, and hence, our study does not reflect the full spectrum of critical COVID-19 patients. Fourth, we had data of only a 3-month CMR examination, and thus, a long-time follow-up is essential to determine the progression or regression of cardiac involvement. Fifth, we did not have native T1 of the healthy control group, which would be useful information to compare with the COVID-19 patients. However, we provide reference normal T1 value base on the previous studies. Sixth, we did not have information regarding hemodynamic instability or evidence of cardiac involvement during initial diagnosis or hospitalization on all patients, which would be helpful to correlate those kinds of patients with the CMR findings.

## Conclusions

Myocardium injury is present in nearly a third of COVID-19 patients at 3 months with evidence of impaired LV GCS and RV GCS and GLS. CMR may be useful to monitor the COVID-19-induced myocarditis progress and remains to be examined.

## Data Availability

The datasets used and/or analyzed during the current study are available from the corresponding author on reasonable request.

## Abbreviations

ACEI: Angiotensin-converting enzyme inhibitor
ARB: Angiotensin receptor blocker
ARDS: Acute respiratory distress syndrome
BSA: Body surface area
bSSFP: Balanced steady state-free precession
CI: Cardiac index
CMR: Cardiovascular magnetic resonance
CO: Cardiac output
COVID-19: Coronavirus disease 2019
CPK: Creatine phosphokinase
CRP: C-reactive protein
ECG: Electrocardiogram
EDV: End-diastolic volume
EF: Ejection fraction
ESV: End-systolic volume
FA: Flip angle
FOV: Field of view
GCS: Global circumferential strain
GLS: Global longitudinal strain
GRS: Global radial strain
IQR: Interquartile range
LGE: Late gadolinium enhancement
LV: Left ventricle/left ventricular
MOLLI: Modified look-locker inversion recovery
MYO: Myohemoglobin
PSIR: Phase-sensitive inversion-recovery
RV: Right ventricle/right ventricular
SI: Signal intensity
SV: Stroke volume
T2w: T2 weighted
TE: Echo time
TnI: Troponin I
TR: Repetition time
TSE: Turbo spin echo
